# Golden years happiness: analyzing the nostalgic aspect of leisure

**DOI:** 10.3389/fpsyg.2024.1466626

**Published:** 2024-09-24

**Authors:** Levent Onal, Fatih Bedir, Murat Turan, Muhammet Mavibas, Buğra Çağatay Savaş, Fatih Yasarturk

**Affiliations:** ^1^Atatürk University, Erzurum, Türkiye; ^2^Erzurum Technical University, Yakutiye, Türkiye; ^3^Bartin University, Bartın, Türkiye

**Keywords:** older adults, retirement, leisure attitude, nostalgia, happiness

## Abstract

Retirees face numerous challenges, particularly in countries with lower socio-economic conditions. Perceived social isolation and happiness levels are seen as significant factors that significantly affect the quality of life in old age. Perceived social happiness levels can disrupt the quality of life in old age and contribute to mental disorders. Recent studies on leisure have suggested a direct impact of individuals’ leisure attitudes on happiness. In this context, leisure nostalgia stands out in the relationship between leisure attitude and happiness levels among retirees. In this study, structural equation modeling (SEM) was employed to examine the relationship between leisure attitude, leisure nostalgia, and happiness levels in a sample of 210 retirees. The findings revealed that leisure nostalgia fully mediated the relationship between leisure attitude and happiness. It was concluded that previous experiences significantly shape retirement, and leisure attitude offers valuable opportunities for enhancing happiness through effective leisure utilization.

## Introduction

Retirement represents a significant life transition, marking the withdrawal from active employment and the conclusion of one’s career (Coe and Zamarro, 2011; Kolodziej and García-Gómez, 2019). This period, often coinciding with old age, offers individuals the opportunity to relax and dedicate more time to leisure activities after years of work (Genoe et al., 2022; Henning et al., 2021). However, retirement introduces new challenges as individuals transition from a structured work routine to a more flexible lifestyle (Beutell and Schneer, 2021). While retirement provides more leisure time (Eismann et al., 2019), it can May disrupt well-being (Asebedo et al., 2019; Cai et al., 2020; Fleischmann et al., 2020).

Retirement is a distinct life stage during which individuals often set new goals and seek novel experiences (Stara et al., 2020). Nevertheless, it can also be a period marked by feelings of emptiness, purposelessness, or social isolation for some retirees (Guo et al., 2021). Therefore, maintaining a fulfilling lifestyle during retirement involves preserving social connections, staying physically active, making informed lifestyle choices, and optimizing leisure time. Indeed, leisure activities play a crucial role during this phase, allowing retirees to explore their interests, engage in hobbies, travel, partake in artistic or athletic pursuits, volunteer, or acquire new skills (Alwajud-Adewusi, 2021; Carney et al., 2021). These leisure pursuits have the potential to significantly enhance retirees’ overall quality of life, increase their levels of happiness, and strengthen their social bonds.

In this context, the concept of leisure attitude assumes paramount importance. Leisure attitude encompasses individuals’ perceptions of their leisure time, their approach to leisure activities, and the significance they attribute to these activities (Belo et al., 2020; Fan and Luo, 2021; Ragheb and Beard, 1982; Önal and Bedir, 2023). It reflects individuals’ attitudes and behaviors toward their leisure time, how they organize and engage in activities, and their overall disposition toward leisure (Kim et al., 2022; Nootens et al., 2019). Allocating time to a hobby or activity that is important to an individual can help them spend their leisure time in a more positive and meaningful way. Additionally, maintaining a balance across various domains of life and engaging in diverse activities also influences leisure attitude (Yoon et al., 2020). It has been stated that the attitude toward leisure time is manifested in three dimensions: cognitive, affective (emotional) and behavioral. The cognitive dimension refers to the stage where individuals’ thought structures are formed, the emotional dimension to the stage where these cognitive structures are transformed into emotions, and the behavioral dimension to the stage where the actions are performed (Kaya et al., 2015). There are many factors that could potentially interact with attitudes toward participation in leisure activities, and it is believed that leisure nostalgia May have a significant impact (Cho, 2020).

**Figure 1:**
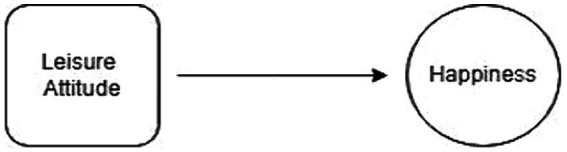
Effect of leisure attitude on happiness (Model 1).

*Hypothesis 1 (H1)*: It is hypothesized that individuals with a positive leisure attitude, characterized by a propensity for engaging in active and meaningful leisure activities, will report higher levels of happiness during retirement (Figure 1).

Leisure attitude is influenced by various factors, including the alignment of activities with personal interests and values, maintaining balance across multiple life domains, effective time management, and active engagement and mindfulness during leisure experiences (Stebbins, 2020; Yoon et al., 2020; Wang, 2019). However, one noteworthy factor, particularly prominent among retirees, is leisure nostalgia—the longing individuals feel for their past leisure experiences (Choi and Yoo, 2017).

Leisure nostalgia, characterized by the emotional connection with positive memories of past leisure activities and experiences, becomes more pronounced during the retirement phase (Gammon and Ramshaw, 2021; Cho, 2021; Wang and Xia, 2021). It holds the potential to not only evoke fond memories of past leisure engagements but also to enhance retirees’ perceptions of their current leisure pursuits. Leisure nostalgia has been addressed in the literature in different dimensions ‘experience, socialization, personal identity and group identity’ (Cho et al., 2014). However, Balcı and Yavaş Tez (2019) stated that leisure nostalgia May vary depending on cultural differences and can be addressed in different dimensions.

**Figure 2:**
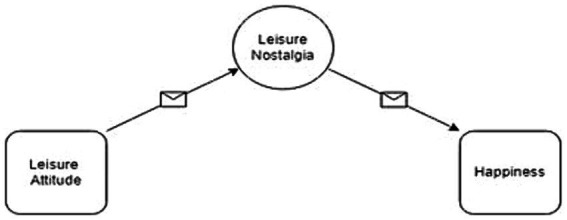
Effect of leisure attitude on happiness through leisure nostalgia (Model 2).

*Hypothesis 2 (H2)*: It is further hypothesized that leisure nostalgia mediates the relationship between leisure attitude and happiness among retirees (Figure 2). Specifically, individuals with a positive leisure attitude who also experience leisure nostalgia are expected to report higher levels of happiness.

Consistent with the hypotheses, there are studies in the literature that support the potential mediating role of leisure nostalgia. FioRito and Routledge (2020) suggest that nostalgia is fundamentally an emotional experience oriented toward the future, enhancing well-being, fostering social connectedness, and deepening perceptions of life’s meaning. Additionally, a good event experience can be associated with greater optimism in the future while creating nostalgic memories (Biskas et al., 2019). Nostalgia has been shown to enhance psychological health and well-being, promoting adaptive functioning, particularly among individuals vulnerable to poor mental health. Compared to merely imagining a desired future experience, nostalgia not only increases well-being but also deepens one’s perception of life’s meaning (Routledge et al., 2012; Routledge et al., 2013).

This study aims to delve deeper into the intricate interplay of leisure attitude and nostalgia, shedding light on their combined impact on happiness and leisure engagement during retirement.

The impact of leisure nostalgia on the attitudes of retirees toward leisure activities cannot be understated (Cho, 2023; Cho et al., 2019). By fostering recollections of past leisure experiences, leisure nostalgia assumes a pivotal role in enhancing the nexus between happiness and one’s outlook on leisure pursuits (Hepper and Dennis, 2022; Layous and Kurtz, 2022; Li et al., 2023; Luo et al., 2022; Zhou et al., 2022). This intricate interplay permits retirees to not only reminisce about their former leisure engagements but also to assess their present leisure pursuits in a more favorable light (Wilson, 2015; Routledge et al., 2013). While the current investigation sheds preliminary light on these dynamics, it is important to emphasize that further comprehensive exploration is indispensable for a deeper comprehension of this intricate relationship. As such, this study endeavors to delve into the ramifications of yearning for past leisure endeavors on both happiness and the disposition toward leisure activities. This avenue of inquiry holds promise in furnishing invaluable insights for the effective harnessing of leisure during the retirement phase.

### Research model

The hypothesized impact of leisure attitudes on happiness in Figure 1 is grounded in the Leisure Motivation Theory (Iso-Ahola, 1980). This theory posits that individuals are motivated to engage in leisure activities due to various intrinsic and extrinsic factors, which fulfill psychological needs and, in turn, contribute to well-being. Positive attitudes toward leisure can lead to greater participation in these activities, thereby enhancing happiness through the satisfaction of personal motivations and the promotion of psychological well-being (Wolfe and Hsu, 2004).

The model presented in Figure 2 hypothesizes that leisure nostalgia May mediate the relationship between leisure attitudes and happiness. This proposition is supported by the Nostalgia Theory, which suggests that recalling positive past experiences, such as satisfying leisure activities, can enhance psychological well-being by fostering feelings of happiness, social connectedness, and a sense of belonging (Routledge et al., 2013). Additionally, grounded in Leisure Motivation Theory (Iso-Ahola, 1980), the model posits that positive leisure attitudes lead to greater participation in leisure activities, which is further amplified through the mediating effect of leisure nostalgia, thereby enhancing overall happiness.

## Materials and methods

The study followed a relational screening model to investigate the relationship between leisure attitude and happiness and leisure nostalgia among retirees at Türkiye. The objective of this study was to investigate the potential mediating role of leisure nostalgia in the relationship under examination. Structural Equation Modeling (SEM) was employed as the statistical technique to analyze the collected data and examine the predictive associations among the variables under investigation (Fraenkel et al., 2012).

### Sample group and data collection process

The universe of this research; retired population living in Erzurum. According to Türkiye İstatistik Kurumu [TÜİK] (2016) data for January 2022, this population constitutes a total of 24 thousand people. The sample of the study was selected by convenient sampling method, which is one of the non-random sampling methods (Gravetter and Forzano, 2015). The sample group consisted of retired individuals residing in the province of Erzurum, Turkey. The questionnaires were administered to retired individuals who spend their time in coffeehouses, cafes, parks, and recreational areas created for community service purposes, representing the sample group. In order to obtain more accurate data, the surveys were completed through face-to-face interviews and on a voluntary basis by the participants (Eysenbach, 2004). In the study, ethical principles outlined in the Helsinki Declaration regarding research involving human subjects, including informed consent, privacy, and procedures, were followed. Prior to answering the questionnaire, participants were provided with information about the purpose of the research (research article) and gave their consent. A total of 210 data were collected without any errors through face-to-face interviews. Of the participants, 71 were female and 139 were male. The completion time for the surveys filled out through the interview method ranged from 6 to 9 min.

### Research model

This study was designed using a correlational research model to examine the effects of leisure attitude and nostalgia on happiness among retired individuals. In this context, the Structural Equation Model (SEM) was utilized to explain the predictive correlations between the variables.

### Data collection instruments

The data collection instruments in the study consist of four sections: a personal information form, “Oxford Happiness Scale,” “Leisure Nostalgia Scale” and “Leisure Attitude Scale.”

### Personal information form

The personal information form, prepared by the researchers, includes questions about participants’ gender, age, marital status, retirement age, and educational background.

### Oxford Happiness Scale

The scale, originally developed by Argyle et al. (1989) and later revised by Hills and Argyle (2002) to create a short form, consists of 29 items. The Turkish adaptation of the scale was conducted by Doğan and Akıncı Çötok (2011). The self-report scale is a five-point Likert-type scale and consists of 7 items. The scale is rated on a scale from “1-Strongly Disagree” to “5-Strongly Agree.” The lowest score is 7, and the highest score is 35. Increasing scores indicate higher levels of happiness. The reliability coefficient of the scale was found 0.74, according to Doğan and Akıncı Çötok (2011). The reliability of the scale used in the study was determined using Cronbach’s Alpha, and the coefficient was calculated to be 0.79.

### Leisure Attitude Scale – short form

The scale, originally developed by Teixeira and Freire (2013) with 18 items and 3 subdimensions (cognitive: 1–6, affective: 7–12, behavioral: 13–18), has been adapted into Turkish by Önal and Bedir (2023). The scale utilizes a 5-point Likert rating. The lowest score is 18, and the highest score is 90. Increasing scores on the scale indicate a higher level of leisure attitude. The reliability coefficient of the scale was found 0.88, according to Önal and Bedir (2023). The reliability of the scale used in the study was determined using Cronbach’s Alpha, and the coefficient was calculated to be 0.84.

### Leisure Nostalgia Scale

Another data collection instrument used in this study is the “Leisure Nostalgia Scale” developed by Cho et al. (2019). The scale consists of 15 items and 3 subdimensions. The Turkish validation and reliability study of the scale was conducted by Balcı and Tez (2019). As a result of the study, the scale was confirmed to consist of 3 subscales and 15 items. The scale utilizes a 7-point Likert rating. The lowest score is 15, and the highest score is 105. The subscales of the scale include spatial memories (5 items), social memories (6 items), and group rituals (4 items). The increase in the scores obtained from the scale indicates that the nostalgic feelings are high. The reliability coefficient of the scale was found 0.78, according to Balcı and Tez (2019). The reliability of the scale used in the study was determined using Cronbach’s Alpha, and the coefficient was calculated to be 0.76.

### Data analysis

To test the hypotheses of the study, Structural Equation Modeling (SEM) was employed. SEM is a collection of statistical techniques that allow for the examination of a series of relationships between one or more independent variables (Ullman and Bentler, 2012). It provides a framework for assessing the relationships among observed variables and latent variables, allowing researchers to investigate complex relationships and evaluate the fit of a proposed model. By utilizing SEM, the study aimed to analyze the hypothesized relationships and gain a deeper understanding of the interrelationships between variables. SEM was chosen for the correlation analysis in this study because it allows for the estimation of parameters of correlation between latent variables and also enables the determination of error variances. The descriptive statistics of the variables were calculated using SPSS 24.0 software, while model testing was conducted using AMOS 24.0 software. The “Maximum Likelihood (ML)” and “Covariance Matrix” were used as parameter estimation methods. These methods are commonly employed in SEM to estimate the model parameters and assess the goodness of fit between the proposed model and the observed data.

First, the normality assumptions of the data was examined to determine whether they met the requirements for constructing the structural equation model. This was done by examining the kurtosis and skewness coefficients. The data set exhibited a normal distribution characteristic. The normality tests of the utilized scales yielded results within the range of values for Happiness (Skewness −1.5 and Kurtosis +1.1), Leisure Attitude (Skewness −1.4 and Kurtosis +1.1), and Leisure Nostalgia (Skewness −1.4 and Kurtosis +1.1), indicating a homogeneous distribution. After testing the normality assumptions, factors such as variance inflation factor and autocorrelation were examined before the analysis. It was found that there was no autocorrelation and the variance inflation factors were within the required limits. Subsequently, it was decided that the data set was suitable for parametric statistical analysis, and the data analysis process commenced.

Common method variance was analyzed with the Harman Single Factor test. According to the Harman test, there is no problem of common method variance when all the expressions are collected in a single factor and there is less than 40% of the variance explained (Podsakoff et al., 2003). The results showed that the factors had an eigenvalue of “LA” and “H,” and the variances explained 33.86%, 29.62%, and 37.58% which were <40%. According to the zero order correlation analysis, the correlation of the marker variable with both variables was found to be significant. Model 1 fit indices as follows: *χ*^2^/df = 2,98; RMSEA = 0.06; CFI = 0.97; GFI = 0.91 and Model 2 fit indices as follows: *χ*^2^/sd = 1.88; CFI = 0.95; GFI = 0.91; RMSEA = 0.05. According to these results, it can be stated that common method variance is not a problem in the study.

## Results

Descriptive statistics were examined for a group of 210 individuals in Table 1. Among this group, 139 individuals (66.2%) were male, and 71 individuals (33.8%) were female. Regarding educational background, 45 individuals (21.4%) had completed primary education, 96 individuals (45.7%) had completed secondary education, 63 individuals (30%) had completed high school, and 6 individuals (2.9%) were university graduates. The majority of individuals in this group had received secondary education. Furthermore, among the participants, 80 individuals (38.1%) were employed in public service, 55 individuals (26.2%) were workers, and 75 individuals (35.7%) were self-employed and had retired. The retirement years ranged from 1 to 15, with an average retirement age of 6.7 years. The age range was between 57 and 80, with an average age of 66.8 years.

**Table 1:** Descriptive of the participants.

	*N*	%
Gender		
Male	139	62.9
Female	71	37.1
Education		
Primary education	45	21.4
Secondary education	96	45.7
High school	63	30.0
Undergraduate	6	2.9
Institution of retirement		
Public servant	80	38.1
Laborer	55	26.2
Self-employment	75	35.7

Table 2 presents the descriptive statistical results of all variables that showed significant correlation with each other in LA, H and LN, and the correlation coefficients between the variables (*p* < 0.01). Table 2 shows that the mean scores for Leisure Nostalgia (LN) at 2.75, Leisure Attitudes (LA) at 2.93, and Happiness (H) at 3.20 suggest moderate levels of nostalgia and positive attitudes toward leisure, with a relatively higher level of overall happiness among participants. These mean values complement the correlation coefficients by providing context to the relationships between these variables.

**Table 2:** The examination of the Pearson product–moment correlation among variables.

	LN	LA	H	X̄	ss
LN	1	0.177^**^	0.408^**^	2.75	0.82
LA		1	0.683^**^	2.93	1.08
H			1	3.20	1.20

**p ≤ 0.01. LN, leisure nostalgia; LA, leisure attitude; H, happiness.

**Figure 3:**
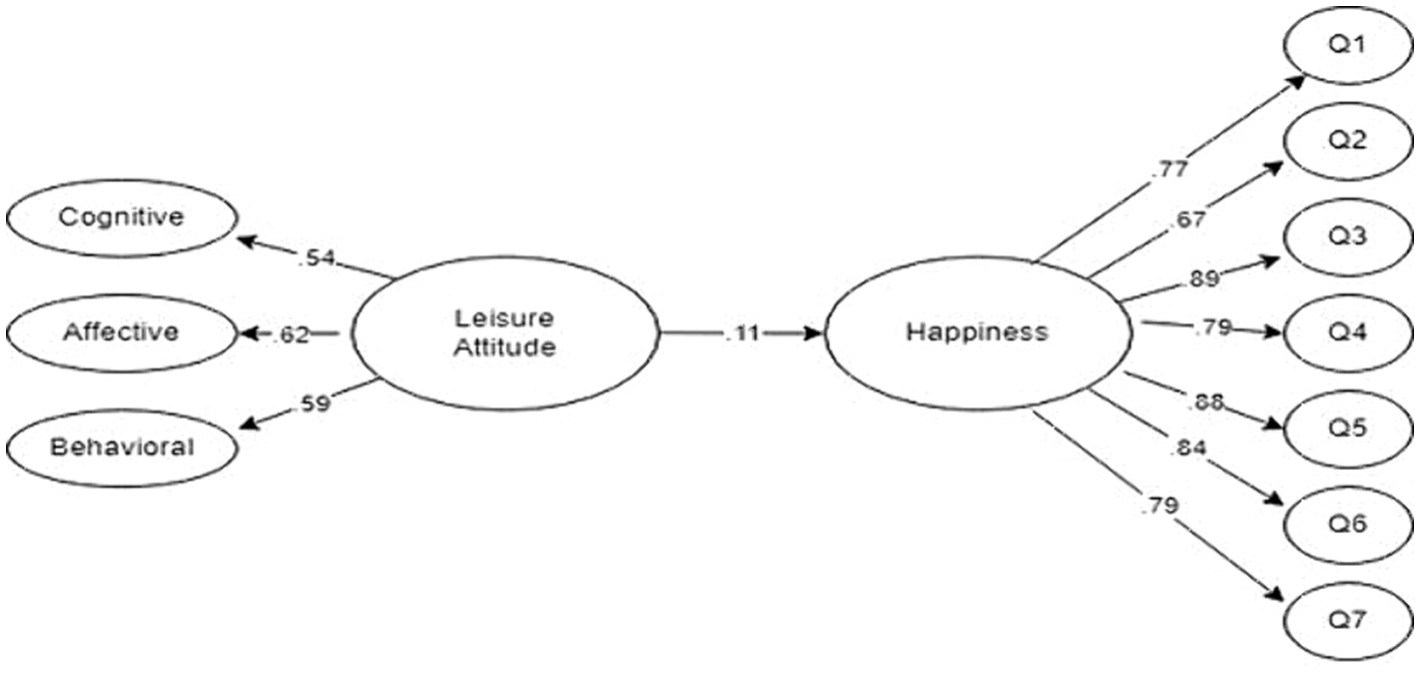
Standardized estimated results of the effect of leisure attitude on happiness (standardized SEM results for Model 1). “Q” represents the items related to the happiness scale.

When assessing the goodness-of-fit measures for the structural model depicted in Figure 3, it becomes apparent that the latent variables in Model 1 demonstrate a statistically significant association with their corresponding observed variables (*p* < 0.01). Results suggest that the structural model exhibits a satisfactory fit in Table 3. Specifically, based on the results of Model 1, it was observed that Leisure Attitude exerted a positive and significant predictive impact on Happiness (*β* = 0.11, *p* < 0.01).

**Table 3:** The fit index values of the model showing the effect of leisure attitude on happiness.

Fit indexes	Acceptable limit	Excellent	Values in the model	Conformity
CMIN/df	Between 2 and 5	≤2	2.98	Acceptable
RMSEA	Between 0.050 and 0.080	Between 0.000 and <0.050	0.06	Acceptable
GFI	0.85 and above	=0.90 and above	0.91	Excellent
AGFI	0.85 and above	=0.90 and above	0.86	Acceptable
CFI	0.95 and above	0.97 and above	0.97	Excellent
RMR	Between 0.050 and 0.080	Between 0.000 and <0.050	0.07	Acceptable
NFI	0.90 and above	0.95 and above	0.92	Acceptable

**Figure 4:**
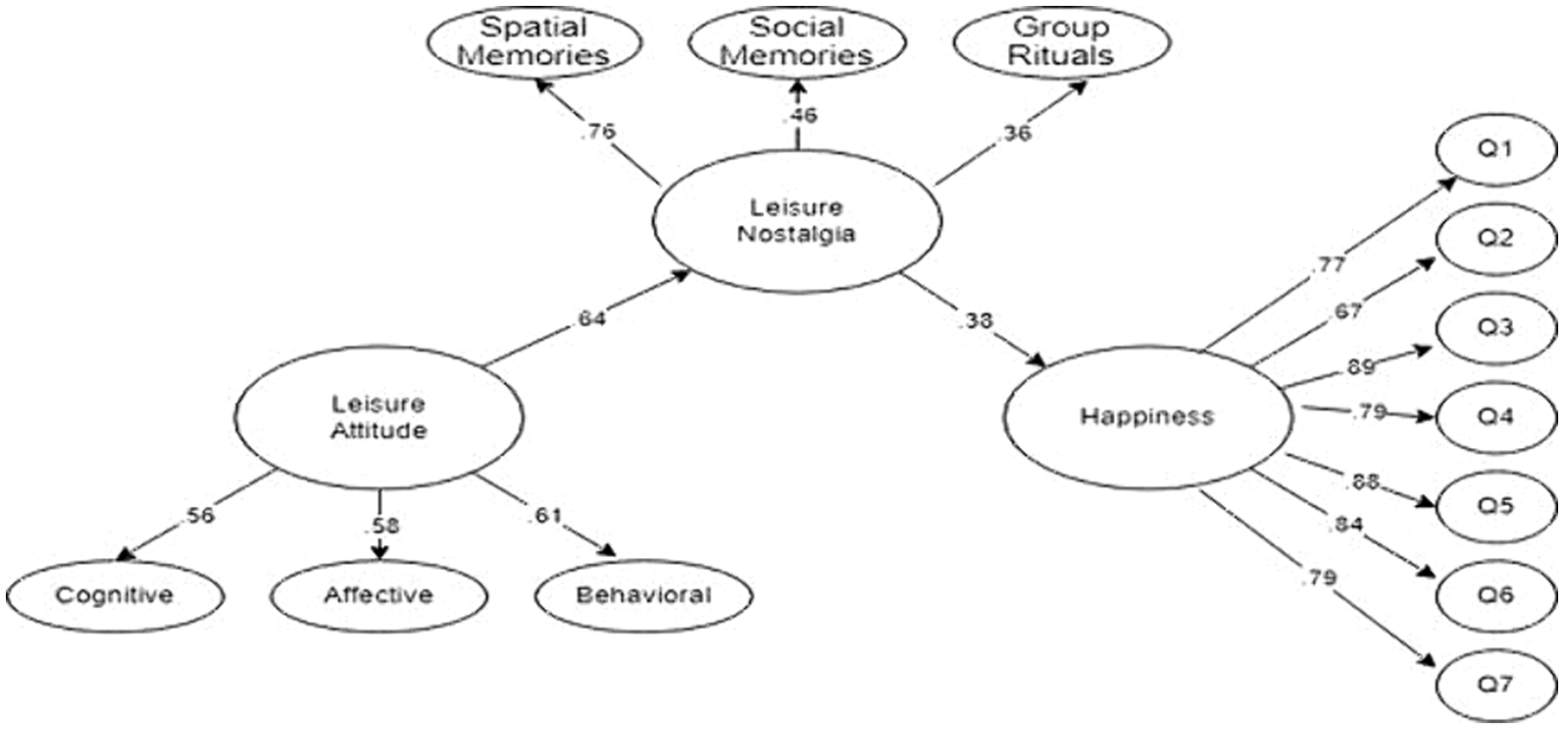
Standardized estimated results demonstrating the effect of leisure nostalgia its mediator role between leisure attitude and happiness (standardized SEM results for Model 2).

Upon assessing the fit indices of the model depicted in Figure 4, it becomes apparent that the latent variables in Model 2 demonstrate a statistically significant association with their corresponding observed variables (*p* < 0.01). These results suggest that the structural model exhibits a satisfactory fit in Table 4. Consequently, the hypothesis proposing that Leisure Nostalgia serves as a complete mediator in the link between Leisure Attitude and Happiness is supported by the empirical findings (*β* = 0.38, *p* < 0.01).

**Table 4:** The fit index values of the model showing the leisure nostalgia, happiness and leisure attitude.

Fit indexes	Acceptable limit	Excellent	Values in the model	Conformity
CMIN/df	Between 2 and 5	≤2	1.88	Excellent
RMSEA	Between 0.050 and 0.080	Between 0.000 and <0.050	0.058	Acceptable
GFI	0.85 and above	=0.90 and above	0.91	Excellent
AGFI	0.85 and above	=0.90 and above	0.86	Acceptable
CFI	0.95 and above	0.97 and above	0.95	Acceptable
RMR	Between 0.050 and 0.080	Between 0.000 and <0.050	0.06	Acceptable
NFI	0.90 and above	0.95 and above	0.96	Excellent

## Discussion

The phenomenon of increasing leisure in human life has been directly associated with individuals’ happiness (Liu and Da, 2020; Nawijn and Veenhoven, 2012). The influence of past experiences on happiness and well-being is also significant (Leunissen et al., 2021). Previous studies in the literature have provided evidence that leisure activities enhance the happiness of retired individuals, and nostalgia for leisure also contributes to increased happiness (Cho, 2021; Cho, 2023; Fludernik, 2020; Lin et al., 2021). Carrasco et al. (2018) and Chaudhry et al. (2024) found that nostalgic ruminations on past roles and activities help individuals reconcile their current selves, expanding understanding of the role of nostalgia in everyday life. While previous research has focused on digital self-representation and its connection to past identities in older adults, our research expands the scope by examining how leisure nostalgia contributes to overall happiness and well-being. This highlights the potential for nostalgic elements to promote psychological health and life satisfaction not only in digital environments but also in leisure activities. These findings suggest that integrating nostalgia into leisure interventions May have broad benefits in both digital and real-world contexts. However, in this study, the mediating role of leisure nostalgia was examined, and the impact of leisure attitude on happiness was investigated.

The impact of nostalgia for past experiences during retirement on leisure attitude holds insights for younger individuals to feel good and happy in their old age. In this context, the research aims to determine the mediating role of leisure nostalgia in the relationship between leisure attitude and happiness. The influence of the predictor variables on the levels of happiness among retired individuals can be discussed in two ways. The direct effect of leisure attitude on happiness can be considered as one aspect (Model I), (*β* = 0.11, *p* < 0.01). The indirect effect, on the other hand, can be examined by considering the mediating role of leisure nostalgia in the relationship between leisure attitude and happiness (Model II), (*β* = 0.38, *p* < 0.01). Fit indices obtained as a result of testing measurement models indicate an adequate fit (Tabachnick and Fidel, 2007).

In psychology, attitude is expressed as a state of emotion, and a positive emotional state is important for achievement and happiness (Kamthan et al., 2019). Some studies in the literature have found that leisure activities enhance the happiness of retired individuals (Kim and Moen, 2002; Lachman and Agrigoroaei, 2010). In a study conducted by Lachman and Agrigoroaei (2010), it was found that individuals who engage in leisure activities have higher happiness. Furthermore, in their study, Kim and Moen (2002) found a link between participation in leisure activities during retirement and individuals’ increased sense of happiness. However, in a study conducted by Gorry et al. (2018), it was found that the impact of leisure activity participation on retired individuals’ happiness was limited. This May be due to cultural and economic differences. This discrepancy in findings suggests the need for further research on how leisure activities affect the happiness of retired individuals.

Initially, we identified that the leisure attitude of retired individuals influenced their levels of happiness (0.11). However, when the mediating variable of leisure nostalgia was added to the model, it was observed that the effect of leisure attitude on happiness increased to 0.38. The findings indicate that leisure attitude is an important factor in determining the levels of happiness among retired individuals. Additionally, it has been determined that the emotion of nostalgia plays a mediating role in happiness and strengthens the relationship between leisure attitude and happiness. Similar studies can be found in the literature (Lu et al., 2022; Cho, 2023; Cho et al., 2021). In their study, Lu et al. (2022) found that leisure attitude have a positive impact on the happiness of retired individuals. The study also demonstrated that leisure nostalgia acts as a mediating variable, altering the influence of leisure attitude on happiness. Cho (2023) and Cho et al. (2021) have found significant results suggesting that leisure nostalgia can be a significant predictor of happiness, shedding light on future studies. In a study conducted by Zhang (2023), the relationship between leisure attitude, leisure nostalgia, and subjective well-being was examined. According to Stone and Mackie (2013), they stated that there is a close relationship between subjective well-being and happiness and can express a common point. Therefore, the findings revealed a positive relationship between leisure attitude, leisure nostalgia and happiness. Cho (2021) conducted a study focusing on older adults, investigating the relationship between leisure attitude, leisure nostalgia, and happiness. The findings indicated that leisure nostalgia mediated the relationship between leisure attitude and happiness. However, it is important to note that there are also studies with differing findings on this topic. Lee and Tideswell (2005) conducted a study among older adults in Korea to examine the effects of leisure attitude and leisure nostalgia on happiness. The findings revealed that leisure attitude had a positive impact on happiness, while the effect of leisure nostalgia was statistically insignificant. These results suggest that the influence of leisure nostalgia on happiness May vary in different contexts or populations. Spiers and Walker (2008) conducted a study among retired individuals to examine the effects of leisure activities and leisure satisfaction on happiness. Riddick and Stewart (1994) investigated the impact of leisure attitudes on happiness among older adults and found that it was not directly related to happiness but was associated with social support. The findings of these studies revealed that leisure activities had a positive influence on happiness, while the effect of leisure satisfaction was statistically insignificant. The reasons for these differences May stem from various factors such as the methodologies employed in the studies, sample characteristics, measurement instruments, age ranges, socio-economic statuses, or cultural variations. Speculation about the underlying causes of these differences requires further research. Therefore, it is crucial for future studies to be designed in a more comprehensive manner, considering different factors. This would contribute to a better understanding of the topic and provide more insights into the relationship between leisure, nostalgia, and happiness among retired individuals.

## Conclusion

In conclusion, the direct effect of leisure attitude on happiness was found to be 0.11 (Model 1), while examining the mediating role of leisure nostalgia in Model 2 revealed that the level of happiness increased to 0.38. This indicates that leisure nostalgia acts as an intermediary variable between leisure attitude and happiness. The findings suggest that leisure nostalgia enhances the impact of leisure attitude on happiness, especially in the retired population, and therefore plays a significant mediating role in happiness levels.

Beyond these findings, this research contributes to the theoretical understanding of how leisure nostalgia functions as a mechanism that bridges past experiences with present well-being. The study advances our knowledge of the psychological processes involved in leisure, highlighting the importance of integrating nostalgic elements in leisure activities for boosting psychological health and life satisfaction.

From a practical perspective, the results have implications for designing leisure programs and social interventions for both older and younger populations. For young individuals, actively engaging in enjoyable leisure activities not only enhances current happiness but also helps accumulate memories that could evoke nostalgia in later life, contributing to future happiness. For retirees, promoting activities that evoke leisure nostalgia can be a powerful tool to increase their overall life satisfaction and well-being. This emphasizes the importance of creating structured leisure environments that encourage reflection on positive past experiences as a means to improve emotional well-being.

### Limitations and suggestions

To enhance the happiness levels of retired individuals, it is beneficial to provide tips on positively influencing their leisure attitudes and maintaining the feeling of nostalgia. Specifically, encouraging retired individuals to revive their past memories during their leisure, intensifying the nostalgic impact of those memories, and thereby increasing their happiness levels can be suggested. Exactly, preserving positive memories of past leisure experiences can be recommended as a means to increase happiness levels. Encouraging retired individuals to actively reminisce about their past leisure activities and cherish the positive memories associated with them can contribute to their overall sense of well-being and happiness.

Indeed, implementing strategies that enhance the ability of retired individuals to remember and revive their past leisure experiences can leverage the impact of leisure attitude on happiness. By focusing on developing skills related to reminiscing and revitalizing past leisure activities, it is possible to enhance the influence of leisure nostalgia on happiness and ultimately increase the overall well-being of retired individuals.

## Data Availability

Publicly available datasets were analyzed in this study. This data can be found: https://doi.org/10.48623/aperta.252437.
